# Measurement Invariance of the Brief Resilient Coping Scale (BRCS) in Peruvian and Spanish Older Adults

**DOI:** 10.1007/s10823-021-09441-z

**Published:** 2021-11-08

**Authors:** Jose M. Tomas, Tomás Caycho-Rodríguez, José Ventura-León, Patricia Sancho, Cirilo H. García, Walter L. Arias

**Affiliations:** 1 Universidad de Valencia, Valencia, Spain; 2 Universidad Privada del Norte, Lima, Peru; 3 Universidad Autónoma de Nuevo León, San Nicolás de los Garza, Mexico; 4 Universidad Católica San Pablo, Arequipa, Peru

**Keywords:** Older adults, Factorial invariance, Resilience

## Abstract

Although the Brief Resilient Coping Scale (BRCS) has been validated in some European and American countries, there are no studies that evaluate its factorial invariance among different nations. In this sense, the objective of the study is to evaluate the factorial invariance of the BRCS in samples of older adults in Peru and Spain, using multigroup Confirmatory Factor Analysis. 236 older adults from Peru participated (Mean age = 72.8, SD = 6.90) and 133 older adults from Spain (Mean age = 71, SD = 7). In the Peruvian sample 78.4% were women and 21.6% men; while in the Spanish sample the majority were women (69.9%). The BRCS was scalar invariant but not strictly invariant between Spain and Peru. Our results found invariance of the structure, factor loadings and intercepts in both countries. These results support the use of BRCS in studies that compare the resilience between samples of older adults in both countries, and encourage applied research for the development of resilience in older adults in Spain and Peru.

## Introduction

In recent years, resilience has received attention from the scientific community as an important part of successful aging (Felten & Hall, 2001; Jopp & Smith, 2006; Resnick, 2014). Although different theoretical perspectives coexist on resilience during the old age (Cosco et al., 2017; Fernandes de Araújo et al., 2015; Windle et al., 2008), commonly resilience is understood as the set of personal and contextual resources that enable individuals to successfully cope and adapt to the various stressors that appear throughout life (Luthar & Cichetti, 2000; Masten, 2007). This conceptualization considers resilience as a protective self-regulating mechanism (Hardy et al., 2004; Masten, 2007; Sojo & Guarino, 2011) in the face of stressful situations in the old age, such as the gradual loss of autonomy, cognitive impairment, lack of mobility, frailty, economic uncertainty, or dealing with significant others' death as well as his/her own death (Aldwin & Igarashi, 2012; Fried et al., 2004; Grenier, 2005; Nygren et al., 2005; Ryff et al., 1998; Serrano-Parra et al., 2012; Smith & Hayslip, 2012). Therefore, some authors consider that resilience in the older adults allows the optimization of personal resources like prosocial behaviors, self-esteem, spirituality, sense of humor, creativity, positive attitude, flexibility, self-determination, or purpose in life (Wild et al., 2011; Ebner et al., 2006; Gattuso, 2003; Hardy et al., 2004; Ong et al., 2009; Resnick, 2014; Serrano-Parra et al., 2012).

Empirical evidence points out that resilience is a predictor of perceived health and wellbeing in old people even in the face of disease and adversity (Davydov et al., 2010; Lamond et al., 2008; Wiles et al., 2012) and this independently of social status (Wild et al., 2011). Therefore, the identification of resilient strategies during the life span is important for the implementation of interventions to promote mental health (Wahlbeck, 2015). An adequate measurement of resilience in the older adults is needed and important both for clinical practice and research (Resnick & Inguito, 2011).

The Brief Resilient Coping Scale (BRCS) is one of the scales used to measure resilience among the many available (for a review see Resnick, 2014). The BRCS is a short unidimensional scale that aims to assess people's ability to cope with stress adaptively, and it is of easy application and interpretation. The Spanish version of the BRCS has been extensively used in research with older adults in Spain (Perez-Blasco et al., 2016; Sales et al., 2015; Tomás et al., 2012a, 2012b). It has shown evidence of validity and reliability in samples of older adults in Spain (Navarro-Pardo et al., 2015; Tomás et al., 2012a, 2012b), in Peru (Caycho-Rodríguez et al., 2018), and also in its Portuguese versión in Portugal (Belo et al., 2016).

Assessing protective factors such as resilience can be a major challenge as they can vary by age group, different life circumstances, as well as between different countries and cultures (Hjemdal et al., 2015). Although BRCS has demonstrated good psychometric properties in older adults from different countries, its intercultural validity has not been evaluated, which is important since the meaning of resilience can vary according to different cultural contexts. There is no evidence on the measurement invariance of the Spanish version of the BRCS across Latin American and Spanish countries.

This measurement invariance is needed in order to make meaningful cross-cultural comparison among older adults in these countries (Byrne & Stewart, 2006). Measurement invariance is a key procedure for studies that compare two or more groups (gender, age, marital status, countries, cultures, etc.) because it tests the equivalence of the meaning of the items between the compared groups (Byrne, 2008; Inglés et al., 2008; Schoot et al., 2012). If the instrument shows a lack of invariance, then the comparisons between the groups are partial and not significant (Pedraza & Mungas, 2008), and the validity of empirical conclusions are not granted (Byrne, 2008). Therefore, cross-cultural comparisons are only possible if there is empirical evidence for measurement invariance (Taylor, 2013; Van der Schoot et al., 2012).

Currently, the evidence on the cultural factors that contextualize how resilience is defined and expressed on day to day in different populations is scarce, and accordingly the cross-cultural validation is absent (Boyden & Mann, 2005; Ungar, 2008). Likewise, the influence of age on resilience in different cultures has not been adequately demonstrated (Schönfeld et al., 2017). The absence of measurement invariance studies is not limited only to resilience, as the invariance in different psychological constructs has not been sufficiently analyzed either (Bieda et al., 2016; Borsboom, 2006). With all the aforementioned in mind, the research aim was: Is the BRCS factorially invariant in Peruvian and Spanish older adults’ samples?

## Method

### Sample and Procedure

#### Peruvian Sample

The Peruvian sample was composed of 236 older adults who were attending to Centers for older adults in the Peruvian city of Trujillo. A non-probability sampling for convenience was used based on the following inclusion criteria: (a) minimum age 60 years; (b) without any apparent physical (functional) or mental disability (dementia) and; (c) have given their informed consent. The data was previously used in a BRCS validation study in Peruvian older adults (Caycho-Rodríguez et al., 2018). The BRCS application was carried out individually or in small groups of a maximum of three participants. 78.4% of the participants were women and 21.6% were men. Mean age was 72.8 years (Sd = 6.90). Regarding marital status, 1.3% were single, 34.7% were married, 25.8% lived with a partner, 15.7% were divorced, and 22.5% were widows or widowers. 10.6% lived alone, 35.2% lived with their husband or wife, 26.7% lived with sons and/or daughters, 25.4% lived with husband or wife and sons or daughters, and finally 21% lived with other relatives. With respect to quality of life, 55.1% declare good or very good life quality, 39.4% average life quality and 5.5% a bad quality of life. The study protocol in Peru received ethical approval from the Universidad Privada del Norte.

#### Spanish Sample

The Spanish sample was composed of 133 Spanish community-dwelling older adults. The study received University of Valencia’s Ethic Board approval. The sample was recruited in four premises of an Association of elderly people in the City of Valencia (Spain). They were surveyed as part of their participation in formation seminars, and their participation was voluntary. All participants were over 60 years of age, had no apparent physical or mental disability, and gave their informed consent. The participants therefore were a convenience sample. Sample mean age was 71 years and 6 months (SD = 7 years). Most of the sample were women (69.9%). With respect to their educational level, 22.5% had no studies, 60.5% had primary studies, 14.7% studied secondary education, and only a 2.3% had university education. Their marital status was as follows: 66.9% married; 25.6% widows or widowers; 7.6% other status. 93.1% had living sons and/or daughters. Most of them 92.4% lived in their own houses, while the remaining 7.6% were living with their families. 19.1% lived alone in their own house, 56.5% with their partner (usually husband or wife), and 14.5% with other members of the family.

### Instruments

For the purposes of this research, the participants had to answer the BRCS by Sinclair and Wallston (2004). This scale has four indicators highly adaptive and resilient to cope with stress. It was originally validated in a sample of patients with rheumatoid arthritis. The Spanish version was first validated by Tomás et al., 2012a, 2012b) who found that it was a valid and reliable measure of resilient coping.

### Statistical Analyses

Reliability and dimensionality of the BRCS is studied in both samples. Cronbach’s alpha and Composite Reliability Index (CRI) were used to estimate the internal consistency of the scale. Cronbach's alpha is widely used as a measure of internal consistency, but it has several shortcomings, basically that it is only appropriate with essentially tau-equivalent items (and tests), and also that it is a lower bound for the true reliability (Raykov, 2004). An alternative to coefficient alpha is the omega coefficient. Item’s homogeneity was also estimated in the Peruvian and Spanish samples. Alphas, items’ homogeneity and Omegas were calculated with the results of the CFAs in Mplus 8.3 (Muthén & Muthén, 2011).

Dimensionality of the BRCS was analysed with CFAs estimated in Mplus 8.3. Given that samples from two populations (older adults in Peru and Spain) were available, a multi-group or measurement invariance routine was used. The method of estimation chosen has been Weighted Least Squares Mean and Variance corrected (WLSMV). This is the recommended method for ordinal and non-normal variables of five or less categories, as the ones in this study, and it has shown a very good behaviour in simulation studies (Finney & DiStefano, 2013). The invariance routine runs a set of CFAs (Thompson & Green, 2006). First, the theoretical model (one-factor solution) is separately estimated in each sample, and good fit in each sample is established. Second, a multi-group sequence of increasingly constrained CFAs, are estimated and tested (Kline, 2015). This sequence of multi-group models starts with the so-called configural model, that tests for pattern invariance or, in other words, it tests whether or not the same factor structure holds for the two groups simultaneously. If the configural model fits the data, a set of constraints on all factor loadings is imposed. This new multi-group CFA tests for metric or weak measurement invariance. If factor loadings are equal across the two samples, metric invariance holds, which means that respondents in the two samples attribute the same meaning to the latent construct under study. Then, another multigroup CFA with additional constraints on all item intercepts is estimated. This model tests for scalar or strong measurement invariance. If this model fits the data as well as the less constrained models then the meaning of the construct (the factor loadings), and the levels of the underlying items (intercepts) are equal in both groups. Accordingly, groups may be compared on their scores on the factor. Finally, a model with further constraints on all error (uniqueness) variances is estimated. This model tests for strict measurement invariance, although most researchers omit these constraints as not really needed for mean comparisons (Millsap & Olivera-Aguilar, 2012).

The measurement invariance models are nested and their relative plausibility (fit) must be assessed. Their plausibility was assessed using several fit criteria (Kline, 2015): (a) chi-square statistic; (b) the Comparative Fit Index (CFI; Bentler, 1990); the (c) the root mean squared error of approximation (RMSEA); and (d) the Standardized Root Mean Square Residual (SRMR). We have employed the cut-off points for adequate fit proposed by Hu and Bentler (1999) who suggested that a CFI of at least 0.95, a RMSEA less than 0.06 and a SRMR less than 0.08 together would indicate a very good fit of the model to the data. A note of caution is nevertheless needed here. It is well-known that the RMSEA works very poorly when the model evaluated has few degrees of freedom, such as the ones we are testing (Breivik & Olsson, 2001; Kenny et al., 2014). Therefore, RMSEA values were given for completeness, but they cannot really be trusted.

Nested models, as the ones in the invariance routine, can be compared with two rationales (Little, 1997): the statistical and the modeling one. The statistical rationale compares the χ^2^ of the alternative models, with non-significant values suggesting multi-group equivalence or invariance. However, this statistical approach has been criticized, mainly because of too much statistical power (Cheung & Rensvold, 2002). Accordingly, Little (1997), among many others, recommended a modeling approach that uses practical fit indices to determine the overall adequacy of a fitted model. From this rationale, if a parsimonious model (such as the ones that posit invariance) evinces adequate levels of practical fit, then the sets of equivalences are considered a reasonable approximation to the data. Practical fit is usually determined with CFI differences (ΔCFI). CFI differences lower than 0.01 (Cheung & Rensvold, 2002) or 0.05 (Little, 1997) are usually employed as cut-off criteria.

## Results

### Descriptive Statistics and Estimates of Reliability

Table 1 showed means, standard deviations, and measures of skewness and kurtosis of the four indicators in both samples. It also showed the item-total correlation (item homogeneity) for the BRCS items in both samples. All internal consistency estimates at the item level were adequate. Alpha coefficients were high both in Spain (0.83, 95% CI 0.76-0.87) as well as in Peru (0.87, 95% CI 0.84-0.91). Omegas were also high in Spain (0.82, 95% CI 0.75–0.86) and Peru (0.87, 95% CI 0.84–0.91).

**Table 1:** Item content, means, standard deviations, skewness, kurtosis and item-total correlation for the four items in the brief resilient coping scale (BRCS) in both samples

	Peru					Spain				
Item content	Mean	SD	Sk	Ku	*r*_it_	Mean	SD	Sk	Ku	*r*_it_
I look for creative ways to alter difficult situations	3.42	0.96	0.02	− 0.01	0.90	3.45	1.43	− 0.52	− 1.0	0.85
Regardless of what happens to me, I believe I can control my reaction to it	3.62	0.87	− 1.1	1.3	0.87	3.52	1.36	− 0.66	− 0.73	0.85
I believe I can grow in positive ways by dealing with difficult situations	3.50	0.94	− 0.27	0.16	0.88	3.86	1.21	− 0.86	− 0.11	0.78
I actively look for ways to replace the losses I encounter in life	3.78	83	0.92	1.0	0.79	3.85	1.22	− 0.87	− 0.13	0.67

*Sk* Skewness, *Ku* Kurtosis, *r*_*it*_ item-total correlation

### Factorial Validity

In order to explore the dimensionality of the BRCS, two CFAs were estimated and tested separately in the Peruvian and Spanish samples. The model fitted reasonably well in the Peruvian sample: χ^2^ (2) = 9.16, *p* = 0.011, CFI = 0.996, TLI = 0.989, RMSEA = 0.123 [90% CI 0.051–0.209], SRMR = 0.012. Similar results were found for the Spanish sample: χ^2^ (2) = 10.56, *p* = 0.005, CFI = 0.986, TLI = 0.958, RMSEA = 0.182 [90% CI 0.085–0.297], SRMR = 0.026.

### Measurement Invariance

Goodness-of fit indices for the set of measurement invariance models are presented in Table 2. The configural model, which can be considered a baseline model, fitted the data very well, with excellent CFI, TLI and SRMR. Then the weak invariance model (all factor loadings constrained to be equal) was tested, and compared to the configural model. A look at the fit-indices makes clear that factor loadings are invariant across samples. The chi-square difference test was non-significant, and the CFI and RMSEA even improved when factor loadings constraints were added. When all item intercepts were made invariant (strong or scalar invariance), model fit did not deteriorate. Although the chi-square difference test was statistically significant (*p* = 0.005), the impact on practical fit indices was negligible (CFI differences of 0.005) and even some of them improved (the RMSEA was 0.087). Therefore, the hypothesis of strong invariance was retained. Then a model with all errors constrained to equality in both groups was tested. If this model fitted the data as well as the strong invariance, it would be evidence that strict invariance holds. However, the model fit clearly deteriorated, with chi-square differences statistically significant and a clear drop in the practical fit, a CFI difference of 0.071, and larger indices of error. Therefore, the hypothesis of strict invariance was nor supported by the data.

**Table 2:** Goodness-of-fit indices for the set of nested models in the measurement invariance routine

Model	_R_χ^2^	df	*p*	Δ_R_χ^2^	Δdf	*p*	CFI	ΔCFI	TLI	RMSEA	90% CI	SRMR
Configural invariance	19.57	4	< 0.001	–	–		0.994	–	0.982	0.146	0.086–0.214	0.018
Weak invariance	20.77	7	0.004	3.63	3	0.304	0.995	− 0.001	0.991	0.104	0.054–0.157	0.019
Strong invariance	42.90	18	< 0.001	26.27	11	0.005	0.990	0.005	0.994	0.087	0.054–0.121	0.032
Strict invariance	206.08	22	< .001	147.62	4	< 0.001	0.929	0.071	0.961	0.214	0.188–0.241	0.075

^*^p < 0.05, _R_χ^2^ Robust chi-square, *df* degrees of freedom, Δ differences

**Fig. 1:**
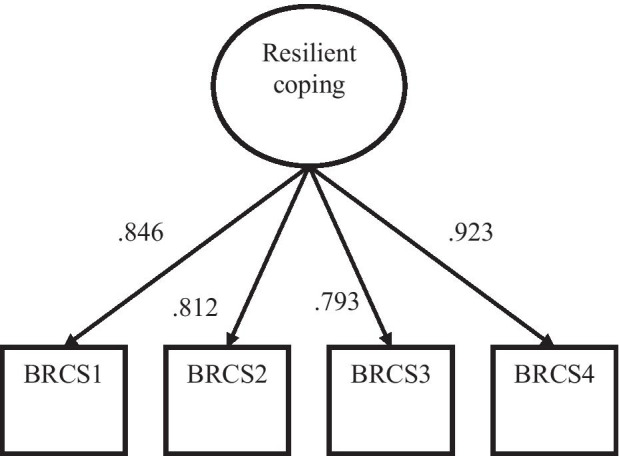
Standardized factor loadings for the four items in the BRCS

Standardized factor loadings are presented in Fig. 1. All items had large relationships with the latent variables. Given that strong invariance holds for these two countries latent means may be compared. Mean difference between the two countries were statistically significant. Spanish older adults had a higher level of resilient coping, although the effect size was relatively low (Mean difference = 0.783, *z* = 2.47, *p* = 0.014, *d* = 0.27).

## Discussion and Conclusions

This research aims to analyze, for the first time, the measurement invariance of the BRCS in older adults from two Spanish-speaking countries, one of them Spain and the other Peru. The results gave support to the scalar invariance in the two countries, but not to strict invariance.

In this sense, the assumptions of equal dimensionality (configural invariance), equivalence of factor loadings (metric invariance) and equal intercepts (scalar invariance) held, results that suggest that the scale works equally well in both Peruvian and Spanish old people samples. In the three models of invariance (configural, metric and scalar), the RMSEA was not within the limits of the cut-off criteria that shows adequate fit. Nevrtheless, this information is not relevant given that in models with small degrees of freedom, as the one we are analyzing, this index does not perform well and should not be used for assessing fit (Byrne, 1998; Kenny et al., 2014; Taasoobshirazi & Wang, 2016). The value of the RMSEA increases as the degrees of freedom and the sample size decrease (Kline, 2015; McCallum et al., 1996) and the other fit indices gave support to scalar invariance. On one hand, it can be concluded that the scale is scalar invariant, an important finding that points out that increment in the level of resilience in the Peruvian sample implies the same increment in the Spanish sample. Or, in other words, the results pointed out that the old people in both countries interpreted the items in the same way (Hjemdal et al., 2015). On the other hand, data did not support strict invariance (equality of item errors). Nevertheless, literature on measurement invariance points out that strict invariance is a very restrictive analysis, and also that if not met it does not compromise the conclusions on sample comparisons (Byrne, 2008). Absence of strict invariance could be associated to cultural, educative, religious, or even perceptual differences with respect to quality of life even among countries who share the same language (Inglehart et al., 2008).

Despite the lack of strict invariance, results admit the presence of a single equivalent factor of resilience, which indicates the absence of differential item functioning in the scale, being an equally accurate measure for the samples of Peruvian and Spanish older adults (Dimitrov, 2010). Thus, the capacity of both samples to cope with stress in an adaptive way configures into a single dimension. Having into account the solid psychometric properties of the BRCS in samples of older adults in different countries (Belo et al., 2016; Caycho et al., 2017; Navarro-Pardo et al., 2015; Tomás et al., 2012a, 2012b), as well as its use in different research on wellbeing and quality of life of the older adults (Pérez-Blasco, et al*.,* 2016; Sales et al., 2015; Tomás et al., 2012a, 2012b), our results allow to consider the BRCS a valid instrument to develop cross-cultural studies on resilience in the Latin American context.

Results may be considered rather provisional, as many other Latin American countries could come into comparison. An adequate interpretation of the results should carefully consider the presence of certain limitations. First, the participants are older adults living in the cities of Trujillo (Peru) and Valencia (Spain), but they belong to convenience samples which are not representative of the older adults’ populations in these countries. Second, the evidence corresponds only to two Spanish-speaking countries. These countries share important cultural characteristics, such as language (Spanish) and religion (Roman Catholic), and therefore other cross-countries measurement invariance studies on the BRCS would be of great interest. This is of particular interest since contextual and economic characteristics have influence on personal resources of the older adults (Lerner et al., 2012). However, there is a need to collect larger samples of different cultural contexts, and to analyze the invariance with respect to other variables, such as gender and age, to better understand the different levels of resilience in all groups. In addition, there is a clear difference between the number of men and women in both countries, where 78.4% and 69.9% of the Peruvian and Spanish participants were women, respectively. In particular, this difference may be important, considering that, in general, women seem to be more resilient than men (MacLeod et al., 2016). The high levels of resilience in older adult women are explained by a better establishment of social connections, seeking support from others, and participation in volunteering and community activities (Kinsel, 2005). However, other studies have not shown conclusive results, as some mention a greater resilience in men (Hirani et al., 2016; Stratta et al., 2013) and others reveal a higher level of resilience in women. (Meng et al., 2019). This lack of consistency between results may be due to social and cultural variations (Meng, et al., 2019). Likewise, the absence of evidence on the invariance of the measurement between genders does not allow us to infer the reasons for these differences. To the best of our knowledge, no study has evaluated the invariance of the BRCS measurement among older adults of both genders. Finally, the number of participants was different in both countries and this could have affected the results of factorial invariance (Yoon & Lai, 2018). Therefore, future research should work with similar sample sizes from different countries to obtain more robust conclusions.

Nevertheless, the evidence shown by this research is sufficient to conclude that the BRCS is a short measure of resilience that has shown good psychometric properties and scalar invariance in the two countries. These results support the use of the BRCS in comparative studies of older adults in Peru and Spain.

## References

[CR1] Aldwin CM, Igarashi H (2012). An ecological model of resiliene in late life. Annual Review of Gerontology and Geriatrics.

[CR2] Belo P, Pocinho R, Rodrigues J (2016). Testing the BRCS structure through a multigroup analysis. Research in Psychology and Behavioral Sciences.

[CR3] Bentler PM (1990). Comparative fit indices in structural models. Psychological Bulletin.

[CR4] Bieda A, Hirschfeld G, Schönfeld P, Brailovskaia J, Zhang XC, Margraf J (2016). Universal happiness? Cross-cultural measurement invariance of scales assessing positive mental health. Psychological Assessment.

[CR5] Borsboom D (2006). When does measurement invariance matter?. Medical Care.

[CR6] Boyden, J., & Mann, G. (2005). Children’s risk, resilience and coping in extreme situations. In: M. Ungar (Ed.), *Pathways to resiliency* (pp. 3–25). Sage. https://us.corwin.com/sites/default/files/upm-binaries/5336_Ungar_I_Proof_Chapter_1.pdf

[CR7] Breivik E, Olsson UH, Cudeck R, Du Toit S, Sörbom D (2001). Adding variables to improve fit: The effect of model size on fit assessment in LISREL. Structural equation modeling: Present and future. A Festschrift in honor of Karl Jöreskog.

[CR8] Byrne BM (1998). Structural equation modelling with LISREL, PRELIS, and SIMPLIS: Basic concepts, applications and programming.

[CR9] Byrne, B. M. (2008). Testing for multigroup equivalence of a measuring in-strument: a walk through the process. *Psicothema*, *20*(4), 872–882. http://www.psicothema.es/pdf/3569.pdf 18940097

[CR10] Byrne BM, Stewart SM (2006). The MACS approach to testing for multigroup invariance of a second-order factor structure: A walk through the process. Structural Equation Modeling.

[CR11] Caycho-Rodríguez T, Ventura-León J, García-Cadena CH, Tomás JM, Domínguez-Vergara J, Daniel L, Arias-Gallegos WL (2018). Evidencias psicométricas de una medida breve de resiliencia en adultos mayores peruanos no institucionalizados. Psychosocial Intervention.

[CR12] Cheung GW, Rensvold RB (2002). Evaluating goodness-of-fit indexes for testing measurement invariance. Structural Equation Modeling: A Multidisciplinary Journal.

[CR13] Cosco TD, Kaushal A, Hardy R, Richards M, Kuh D, Stafford M (2017). Operationalising resilience in longitudinal studies: A systematic review of methodological approaches. Journal of Epidemiology and Community Health.

[CR14] Davydov DM, Stewart R, Ritchie K, Chaudieu I (2010). Resilience and mental health. Clinical Psychology Review.

[CR15] Dimitrov DM (2010). Testing for factorial invariance in the context of construct validation. Measurement and Evaluation in Counseling and Development.

[CR16] Ebner NC, Freund AM, Baltes PB (2006). Developmental changes in personal goal orientation from young to late adulthood: From striving for gains to maintenance and prevention of losses. Psychology and Aging.

[CR17] Felten BS, Hall JM (2001). Conceptualizing resilience in women older than 85: Overcoming adversity from illness or loss. Journal of Gerontological Nursing.

[CR18] Fernandes de Araújo L, Teva I, Bermúdez M (2015). Resiliencia en adultos: Una revisión teórica. Terapia Psicológica.

[CR19] Finney SJ, DiStefano C, Hancock GR, Mueller RO (2013). Nonnormal and categorical data in structural equation modeling. Structural equation modeling: A second course.

[CR20] Fried L, Ferrucci L, Darer J, Williamson J, Anderson G (2004). Untangling the concepts of disability, frailty, and comorbidity: Implications for improved targeting and care. The Journals of Gerontology.

[CR21] Gattuso S (2003). Becoming a wise old woman: Resilience and wellness in later life. Health Sociology Review.

[CR22] Grenier AM (2005). The contextual and social locations of older women’s experiences of disability and decline. Journal of Aging Studies.

[CR23] Hardy SE, Concato J, Gill TM (2004). Resilience of community-dwelling older persons. Journal of the American Geriatrics Society.

[CR24] Hirani S, Lasiuk G, Hegadoren K (2016). The intersection of gender and resilience. Journal of Psychiatric and Mental Health Nursing..

[CR25] Hjemdal O, Roazzi A, Dias MG, Friborg O (2015). The cross-cultural validity of the resilience scale for adults: A comparison between Norway and Brazil. BMC Psychology.

[CR26] Hu L, Bentler PM (1999). Cut-off criteria for fit indexes in covariance structure analysis: Conventional criteria versus new alternatives. Structural Equation Modeling.

[CR27] Inglehart R, Foa R, Peterson C, Welzel C (2008). Development, freedom, and rising happiness: A global perspective (1981–2007). Psychological Science.

[CR28] Inglés, C. J., Marzo, J. C., Hidalgo, M. D., Zhou, X., & Garcia-Fernandez, J. M. (2008). Factorial invariance of the questionnaire about interpersonal difficulties for adolescents across Spanish and Chinese adolescent samples. Measurement and Evaluation in Counseling and Development, 41(2), 89–103. 10.1080/07481756.2008.11909824

[CR29] Jopp D, Smith J (2006). Resources and life-management strategies as determinants of successful aging: On the protective effect of selection, optimization, and compensation. Psychology and Aging.

[CR30] Kenny DA, Kaniskan B, McCoach B (2014). The performance of RMSEA in models with small degrees of freedom. Sociological Research Methods.

[CR31] Kinsel B (2005). Resilience as adaptation in older women. Journal of Women & Aging.

[CR32] Kline RB (2015). Principles and practice of structural equation modeling.

[CR33] Lamond AJ, Depp CA, Allison M, Langer R, Reichstadt J, Moore DJ, Golshanc S, Ganiatsd TG, Jestebce DV (2008). Measurement and predictors of resilience among community-dwelling older women. Journal of Psychiatric Research.

[CR34] Lerner RM, Weiner MB, Arbeit MR, Chase PA, Agans JP, Schmid KL, Warren AA, Hayslip BJ, Smith GC (2012). Resilience across the lifespan. Annual review of gerontology and geriatrics, emerging perspectives on resilience in adulthood and later life.

[CR35] Little TD (1997). Mean and covariance structures (MACS) analyses of cross-cultural data: Practical and theoretical issues. Multivariate Behavioral Research.

[CR36] Luthar, S. S., & Cicchetti, D. (2000). The construct of resilience: Implications for interventions and social policies. *Development and Psychopathology*, *12*(4), 857–885. https://www.ncbi.nlm.nih.gov/pmc/articles/PMC1903337/10.1017/s0954579400004156 PMC1903337 11202047

[CR37] MacLeod S, Musich S, Hawkins K, Alsgaard K, Wicker ER (2016). The impact of resilience among older adults. Geriatric Nursing.

[CR38] Masten AS (2007). Resilience in developing systems: Progress and promise as the fourth wave rises. Development and Psychopathology.

[CR39] McCallum RC, Browne MW, Sugawara HM (1996). Power analysis and determination of sample size for covariance structure modeling. Psychological Methods.

[CR40] Meng M, He J, Guan Y, Zhao H, Yi J, Yao S, Li L (2019). Factorial invariance of the 10-item Connor-Davidson resilience scale across gender among Chinese elders. Frontiers in Psychology.

[CR41] Millsap RE, Olivera-Aguilar M, Hoyle RH (2012). Investigating measurement invariance using confirmatory factor analysis. Handbook of structural equation modeling.

[CR42] Muthén LK, Muthén BO (2011). Mplus user's guide.

[CR43] Navarro-Pardo E, Fernández-Muñoz JJ, Vázquez-Martínez A, Vázquez-Molina J, Moret CMT, Civera-Mollá C (2015). Resilience and the aging process: Assessment tools and needs. Procedia-Social and Behavioral Sciences.

[CR44] Nygren B, Alex L, Jonsen E, Gustafson Y, Norberg A, Lundman B (2005). Resilience, sense of coherence, purpose in life and self-transcendence in relation to perceived physical and mental health among the oldest old. Aging & Mental Health.

[CR45] Ong AD, Bergeman CS, Boker SM (2009). Resilience comes of age: Defining features in later adulthood. Journal of Personality.

[CR46] Pedraza O, Mungas D (2008). Measurement in cross-cultural neuropsy-chology. Neuropsychology Review.

[CR47] Perez-Blasco J, Sales A, Meléndez J, Mayordomo T (2016). The effects of mindfulness and self-compassion on improving the capacity to adapt to stress situations in elderly people living in the community. Clinical Gerontologist.

[CR48] Raykov T (2004). Behavioral scale reliability and measurement invariance evaluation using latent variable modeling. Behavioral Therapy.

[CR49] Resnick B (2014). Resilience in older adults. Topics in Geriatric Rehabilitation.

[CR50] Resnick BA, Inguito PL (2011). The resilience scale: Psychometric properties and clinical applicability in older adults. Archives of Psychiatric Nursing.

[CR51] Ryff CD, Singer B, Love GD, Essex MJ, Lomranz J (1998). Resilience in adulthood and later life: Defining features and dynamic processes. Handbook of aging and mental health: An integrative approach.

[CR52] Sales A, Pardo A, Mayordomo T, Satorres-Pons E, Meléndez JC (2015). Efectos de la terapia cognitivo-conductual sobre la depresión en personas mayores institucionalizadas. Revista De Psicopatología y Psicología Clínica.

[CR53] Schönfeld P, Brailovskaia J, Margraf J (2017). Positive and negative mental health across the lifespan: A cross-cultural comparison. International Journal of Clinical and Health Psychology.

[CR54] Van de Schoot R, Lugtig P, Hox J (2012). A checklist for testing measurement invariance. European Journal of Developmental Psychology.

[CR55] Serrano-Parra, M. D., Garrido-Abejar, M., Notario-Pacheco, B., Bartolomé-Gutierrez, R., Solera-Martínez, M., & Martínez-Vizcaíno, V. (2012). Validez de la escala de Resiliencia de Connor-Davidson (CD-RISC) en una población de mayores entre 60 y 75 años. *International Journal of Psychological Research*, *5*(2), 49–57. https://www.redalyc.org/pdf/2990/299025051006.pdf

[CR56] Sinclair VG, Wallston KA (2004). The development and psychometric evaluation of the brief resilient coping scale. Assessment.

[CR57] Smith GC, Hayslip B (2012). Resilience in adulthood and later life: What does it mean and where are we heading?. Annual Review of Gerontology & Geriatrics.

[CR58] Sojo V, Guarino L (2011). Mediated moderation or moderated mediation: Relationship between length of unemployment, resilience, coping and health. The Spanish Journal of Psychology.

[CR59] Stratta P, Capanna C, Patriarca S, de Cataldo S, Bonanni RL, Riccardi I, Rossi A (2013). Resilience in adolescence: Gender differences two years after the earthquake of L’Aquila. Personality and Individual Differences.

[CR60] Taasoobshirazi, G. & Wang, S. (2016). The performance of the SRMR, RMSEA, CFI, and TLI: An examination of sample size, path size, and degrees of freedom. *Journal of Advances in Quantitative Methods, 11,* 31–39. http://jaqm.ro/issues/volume-11,issue-3/pdfs/2_GI_SH_.pdf

[CR61] Taylor CS (2013). Validity and validation.

[CR62] Thompson MS, Green SB, Hancock GR, Mueller RO (2006). Evaluating between-group differences in latent means. Structural equation modeling: A second course.

[CR63] Tomás JM, Meléndez JC, Sancho P, Mayordomo T (2012). Adaptation and initial validation of the BRCS in an elderly Spanish sample. European Journal of Psychological Assessment.

[CR64] Tomás JM, Sancho P, Melendez JC, Mayordomo T (2012). Resilience and coping as predictors of general well-being in the elderly: A structural equation modeling approach. Aging & Mental Health.

[CR65] Ungar M (2008). Resilience across cultures. British Journal of Social Work.

[CR66] Wahlbeck K (2015). Public mental health: The time is ripe for translation of evidence into practice. World Psychiatry.

[CR67] Wild K, Wiles JL, Allen RES (2011). Resilience: Thoughts on the value of the concept for critical gerontology. Aging & Society.

[CR68] Wiles JL, Wild K, Kerse N, Allen RE (2012). Resilience from the point of view of older people: There's still life beyond a funny knee. Social Science & Medicine.

[CR69] Windle G, Markland DA, Woods RT (2008). Examination of a theoretical model of psychological resilience in older age. Aging & Mental Health.

[CR70] Yoon M, Lai MH (2018). Testing factorial invariance with unbalanced samples. Structural Equation Modeling: A Multidisciplinary Journal.

